# Potential Proteins Interactions with *Bombyx mori* Nucleopolyhedrovirus Revealed by Co-Immunoprecipitation

**DOI:** 10.3390/insects13070575

**Published:** 2022-06-24

**Authors:** Xiong Wang, Guangyu Ma, Feifei Ren, Mian Muhammad Awais, Jingchen Sun

**Affiliations:** Guangdong Provincial Key Laboratory of Agro-Animal Genomics and Molecular Breeding, College of Animal Science, South China Agricultural University, Guangzhou 510642, China; wangttx@outlook.com (X.W.); maguangyu98@163.com (G.M.); zzff43@outlook.com (F.R.); awaismian31@yahoo.com (M.M.A.)

**Keywords:** *Bombyx mori*, nuclear polyhedrosis virus, viral proliferation, co-immunoprecipitation

## Abstract

**Simple Summary:**

The *Bombyx mori* nucleopolyhedrovirus (BmNPV) is a typical model baculovirus, representing one of the major pathogens of the silkworm. We reconstituted the virus in vitro and used it as a bait for immunoprecipitation experiments on cells and silkworm bodies, obtaining a database of proteins potentially interacting with BmNPV. This study provides a protein data reference for the screening of BmNPV receptors and the study of proteins that play a key role in the replication of BmNPV. It is also important to deal with the prevention of BmNPV in silkworms at the molecular level.

**Abstract:**

Virus–host interactions are critical for virus replication, virulence, and pathogenicity. The *Bombyx mori* nucleopolyhedrovirus (BmNPV) is a typical model baculovirus, representing one of the most common and harmful pathogens in sericulture. Herein, we used co-immunoprecipitation to identify candidate proteins with potential interactions with BmNPV. First, a recombinant BV virus particle rBmBV-egfp-p64-3×flag-gp64sp was constructed using a MultiBac baculovirus multigene expression system. Co-immunoprecipitation experiments were then performed with the recombinant BV virus infected with BmN cells and Dazao silkworms. LC-MS/MS analysis revealed a total of 845 and 1368 candidate proteins were obtained from BmN cells and silkworm samples, respectively. Bioinformatics analysis (Gene Ontology, KEGG Pathway) was conducted for selection of proteins with significant enrichment for further confirmation of the effects on BmNPV replication. Overall, the results showed that SEC61 and PIC promoted the replication of BmNPV, while FABP1 inhibited the replication of BmNPV. In summary, this study reveals the potential proteins involved in BmNPV invasion and proliferation in the host and provides a platform for identifying the potential receptor proteins of BmNPV.

## 1. Introduction

Baculovirus is a class of double-stranded circular DNA viruses with a capsid structure and a genome size of 80–180 kb, for whom the main hosts are arthropods [1,2]. Based on the morphology of the inclusion bodies, baculoviruses are divided into two main groups, the nucleopolyhedrovirus (NPV) and the granulovirus (GV) [3,4]. The *Bombyx mori* nucleopolyhedrovirus (BmNPV) is a typical model baculovirus, representing one of the most common and harmful pathogens in sericulture [5,6,7,8]. When BmNPV infects a host, it produces two types of viral particles: the early budded virions (BV) [9], which spread mainly between cells, and the later occlusion-derived virions (ODV), which spread mainly between hosts [10,11,12]. ODV virus particles are packaged in polyhedra of highly symmetric covalent cross-linked lattices [13,14]. BmNPV infects *B.* *mori* larvae mainly through the oral cavity. Polyhedra are alkaline lysed by the host intestinal environment and the membranes of the intestine are disrupted by the virus to form pores [15,16,17]. The nucleocapsid protein of the virus enters the columnar epithelial cells of the host midgut through envelope-mediated membrane fusion and initiates primary infection. The nucleocapsid protein enters the nucleus under the traction of actin, starts transcription, and completes the assembly of the progeny viral nucleocapsid in the nucleus [18,19]. The mature progeny nucleocapsid enters the cytoplasm through the nuclear pore, acquires the host cell membrane structure as the capsule membrane under the traction of the capsid protein, completes the outgrowth process, and forms a new progeny virus. At the late stages of infection, the progeny ODVs are re-embedded into polyhedra and released into the environment following death and disintegration of the host [20].

Virus invasion and replication are inevitably accompanied by interaction with host proteins [21,22,23]. Proper folding of viral and cellular proteins is essential for their function and stability, a process that is usually mediated through cellular molecular chaperones. The invasion and replication mechanisms of BmNPV remain largely unknown, and in particular, the investigation of its receptor protein is warranted. Recently, some proteins were shown to be associated with the invasion and replication of BmNPV, including TRAP1 protein, a member of the heat shock protein 90 family [24], E3 ubiquitin-protein ligase SINA-like 10 (SINAL10) [25], *B. mori* nuclear hormone receptor 96 (BmNHR96) [26], and *B. mori* receptor expression-enhancing protein (BmREEPa) [27].

In this study, to investigate the host proteins involved in the invasion and replication process of BmNPV, we firstly constructed the recombinant BV virus particle rBmBV-egfp-p64-3×flag-gp64sp using a MultiBac system [28,29]. It was then used to infect BmN cells and Dazao silkworms in a co-immunoprecipitation experiment. Data analysis with data generated by mass spectrometry in the *B. mori* species classification of the protein database Uniprot was conducted, revealing a total of 845 and 1368 candidate proteins were obtained from BmN cells and silkworm samples, respectively. Gene Ontology and KEGG Pathway were performed for selecting proteins with significant enrichment for further confirmation of the effects on BmNPV replication. Our study obtained several proteins associated with BmNPV invasion and replication. It provides a database and methodological reference of candidate proteins for the subsequent screening of receptor proteins in the invasion process of BmNPV and the study of key proteins in the replication process.

## 2. Materials and Methods

### 2.1. Genes, Plasmids, Strains, Cells and Silkworm Strain

Genes: the template of enhanced green fluorescent protein (EGFP) and BmNPV envelope protein GP64. Plasmids: pFBDM. Strain: *Escherichia* *coli* TOP10 and BmSw106-inv were preserved by our laboratory. *B. mori* ovary cell, BmN was maintained at 28 °C in Grace medium supplemented with 10% fetal bovine serum (Gibco), 100 U/mL penicillin, and 100 μg/mL streptomycin (Gibco), and the silkworm strain Dazao was maintained at the laboratory.

### 2.2. Construction of Recombinant Baculovirus

With the primers shown in Table 1, the promoter p64, the capsid protein gene 3×flag-gp64sp of BV virus particles, and the fluorescent reporter gene EGFP were obtained by PCR amplification. The fragments of p64 and plasmid pFBDM were treated by restriction enzymes *Spe*I and *Bam*HI (New England Biolabs, Ipswich, MA, USA) and circularized by T4 ligase (Fermentas, Waltham, MA, USA) at 16 °C. After transformation, the recombinant vector pFBDM-p64 was then obtained by PCR. The fragments of 3×flag-gp64sp and plasmid pFBDM-p64 were further treated by restriction enzymes *Eco*RI and *Sal*I (New England Biolabs) and circularized by T4 ligase (Fermentas) at 16 °C. After transformation, the recombinant vector pFBDM-p64-3×flag-gp64sp was obtained. In addition, the fragments of EGFP and plasmid pFBDM-p64-3×flag-gp64sp were treated by restriction enzymes *Xma*I and *Xho*I (New England Biolabs,) and circularized by T4 ligase (Fermentas) at 16 °C. After transformation, the recombinant vector pFBDM-egfp-p64-3×flag-gp64sp was obtained. The recombinant bacmid rBmSw-egfp-p64-3×flag-gp64sp was obtained by transposition of the vector pFBDM-egfp-p64-3×flag-gp64sp into BmSw106 via the Tn7 transposition site, while the recombinant baculovirus was obtained using the recombinant bacmid rBmSw-egfp-p64-3×flag-gp64sp to infiltrate BmN cells for 3 to 5 days.

### 2.3. Co-Immunoprecipitation

**Figure 1:**
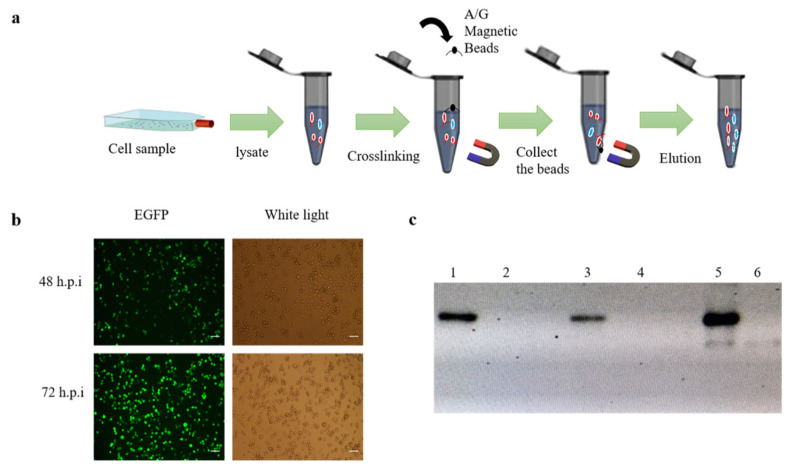
Co-immunoprecipitation of BmN sample. (**a**) Schematic diagram of co-immunoprecipitation of BmN cell. (**b**) Fluorescence image of infection of BmN cells at the stage of sample collection (bar = 100 µm). (**c**) Qualitative analysis of proteins at different stages of immunoprecipitation. (1: Proteins of the lysed cells; 3: Protein not bound to magnetic beads; 5: Protein eluted from the magnetic beads, and 2,4,6 is control).

BmN cells were cultured in 75 cm^2^ cell culture flask and were grown to 70–80%, the recombinant baculovirus rBmBV-egfp-p64-3×flag-gp64sp was added to the flask and incubated for 72 h at 28 °C. Cell samples were then collected using 2 mL PBS. Immunoprecipitation experiments were performed with a Pierce^TM^ Crosslink Magnetic IP/Co-IP kit (Thermo Fisher Scientific, Waltham, MA, USA) (Figure 1a).

**Figure 2:**
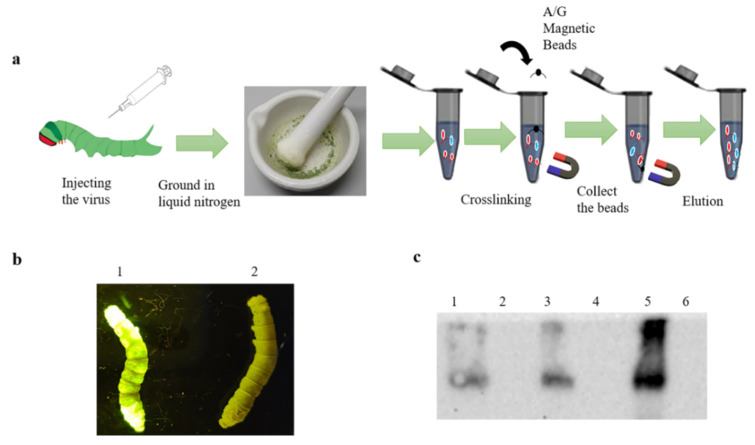
Co-immunoprecipitation of silkworm sample. (**a**) Schematic diagram of co-immunoprecipitation of the silkworm. (**b**) Fluorescence image of infection of silkworm at the stage of sample collection. (**c**) Qualitative analysis of proteins at different stages of immunoprecipitation. (1: Proteins of the lysed silkworm of the epithelium; 3: Protein not bound to magnetic beads; 5: Protein eluted from the magnetic beads, and 2,4,6 is control).

The recombinant baculovirus rBmBV-egfp-p64-3×flag-gp64sp was injected into a Dazao silkworm, and silkworm morbidity was determined by the characteristics of silkworm disease and green fluorescence of silkworm body. The silkworm epithelium is more accessible than other silkworm tissues and less likely to be mixed with proteins from other tissues. Moreover, to ensure the expression of “3×flag-gp64sp”, we chose silkworm epithelium as the experimental sample. Then, the affected silkworm epithelium was collected and ground in liquid nitrogen, and silkworm epithelium samples were collected with 2 mL PBS. Normal silkworms were used as control. Immunoprecipitation experiments were performed with a Pierce^TM^ Crosslink Magnetic IP/Co-IP kit (Thermo Fisher Scientific) (Figure 2a).

### 2.4. Western Blotting

Collected cell samples and silkworm samples were processed by lysate, and after SDS-PAGE, the proteins were transferred into PVDF membranes (Roche Diagnostics, Basel, Switzerland) and incubated with the primary antibody Flag (Beyotime, Suzhou, China), and the secondary antibody, horseradish peroxidase-conjugated goat anti-mouse IgG (Beyotime). The results of Western blotting were analyzed by a ECL Western blotting Detection System (Bio-Rad, Hercules, CA, USA).

### 2.5. Transmission Electron Microscopy

The supernatant of BmN cells infected with the recombinant baculovirus rBmBV-egfp-p64-3×flag-gp64sp was collected and centrifuged at 4 °C for 60 min at 100,000× *g*. The supernatant was discarded, and the precipitate was resuspended with PBS solution and stored at 4 °C. Hydrophilic treatment of copper mesh was conducted. Then, 10 µL of sample was pipetted into the copper mesh and stained with 2% uranium acetate for 45 s. After drying for 2 h, the samples were observed using a transmission electron microscope (Talos F200s, TEM, Thermo Fisher Scientific).

### 2.6. LC-MS/MS Analysis

The sample was loaded onto a preloaded column (3 µm, 120 Å) at a flow rate of 4 µL/min, and were further rinsed and desalted for 5 min. After completion of desalting, the sample was separated by an analytical column with a gradient of mobile phase B from 8 to 38% in 60 min. The ion source spray voltage was set as 2.4 kV, while the atomization air pressure was 12 PSI. In addition, the air curtain air pressure was 35 PSI, while the heating temperature was 150 °C. The sweep time for a single spectrum was 250 ms, and a maximum of 40 secondary profiles with charges of 2^+^ to 4^+^ and counts greater than 260 cps acquired at a time with a cumulative time of 60 ms. Each cycle lasted 2.5 s. The raw data from the mass spectrometry acquisition were processed and analyzed by PEAKS Studio 8.5 software (Thermo Fisher Scientific) with Uniprot (https://www.uniprot.org/, accessed on 5 June 2021) under the *B. mori* species protein database.

### 2.7. Bioinformatic Analysis

The identified proteins were taxonomically annotated by QuickGO (https://www.ebi.ac.uk/QuickGO/, accessed on 8 August 2021). Statistical analysis was performed for Gene Ontology (GO) entries for Biological Process (BP), Cellular Component (CC), and Molecular Function (MF) in which the protein is involved (http://www.geneontology.org, accessed on 8 August 2021).

Pathway enrichment of the candidate proteins identified by LC-MS/MS wasperformed for identifying candidate enriched metabolic pathways with the KEGG database (https://www.kegg.jp/, accessed on 20 September 2021). Statistical analysis was performed for pairs of different types of proteins.

### 2.8. RNAi

With the primers listed in Table 1, T7 RIBO MAX^TM^ expressing the RNAi system kit (Promega, Madison, WI, USA) was used to synthesize the dsRNA (double-strand RNA), and the product was stored at −80 °C. The dsRNA was mixed with the transfection reagent (Promega) and added to BmN cells for incubation at 28 °C. After 24 h.p.i, the recombinant baculovirus BmBV-egfp-p64-3×flag-gp64sp was added. Samples from two timepoints (12 and 24 h) were collected, respectively. Normal BmN cells were used as control.

### 2.9. Quantitative Real-Time PCR (qPCR)

The total RNAs of BmN cells were extracted with the Kit RNA fast 2000 (Fastagen, Shanghai, China) and then reverse transcribed into cDNA using an RT reagent kit with gDNA Eraser (TaKaRa, Shiga, Japan). qPCR was further performed with the primers listed in Table 1, and Ribosomal protein RP49 was used as an internal gene control. The qPCR was carried out in a 20 μL reaction mixture that contained 0.8 μL of cDNA, 0.5 mM of each specific primer, and 10 μL of 2xiTaq^TM^ Universal SYBR Green Supermix (Bio-Rad), and each test was conducted in triplicate. The reaction conditions were set as 95 °C for 5 min, followed by 40 cycles at 95 °C for 15 s and 60 °C for 35 s.

## 3. Results

### 3.1. Construction of Recombinant Baculovirus BmBV-egfp-p64-3×flag-gp64sp

**Figure 3:**
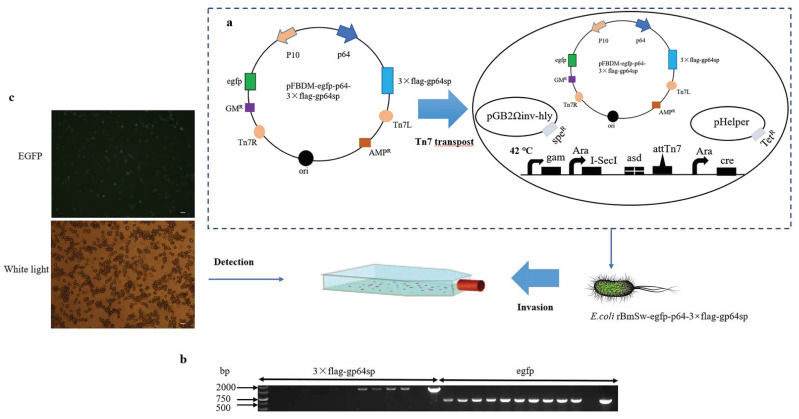
Schematic diagram of recombinant baculovirus construction. (**a**) Transfer vector to *E. coli* BmSw106 via Tn7 transfer site to construct new recombinant *E. coli* rBmSw106-egfp-p64. (**b**) Determination of the success of recombinant *E. coli* rBmSw106-egfp-p64 construction by PCR detection of *gp64* and *egfp* genes. (**c**) Detection of primary recombinant baculovirus BmBV-egfp-p64-3×flag-gp64sp production by inverted fluorescence microscope (bar = 100 µm).

The successfully constructed transfer vector pFBDM- egfp-p64-3×flag-gp64sp was transferred into strain BmSw106 by Tn7 transfer site and screened on a solid medium LB containing Kanamycin (Kan), Spectinomycin (Spe), Tetracycline (Tet), Gentamicin (Gm), and Heptanedioic acid,2,6-diamino (DAP). The positive-strain rBmSw106- egfp-p64-3×flag-gp64sp was identified by PCR (Figure 3a). The PCR results indicated that the recombinant bacterium rBmSw106-egfp-p64-3×flag-gp64sp was successfully constructed (Figure 3b). Subsequently, BmN cells were infected using the recombinant strain rBmSw106-egfp-p64-3×flag-gp64sp. In addition, the recombinant baculovirus BmBV-egfp-p64-3×flag-gp64sp was determined by identifying the green fluorescence by inverted fluorescence microscopy (Figure 3c).

**Figure 4:**
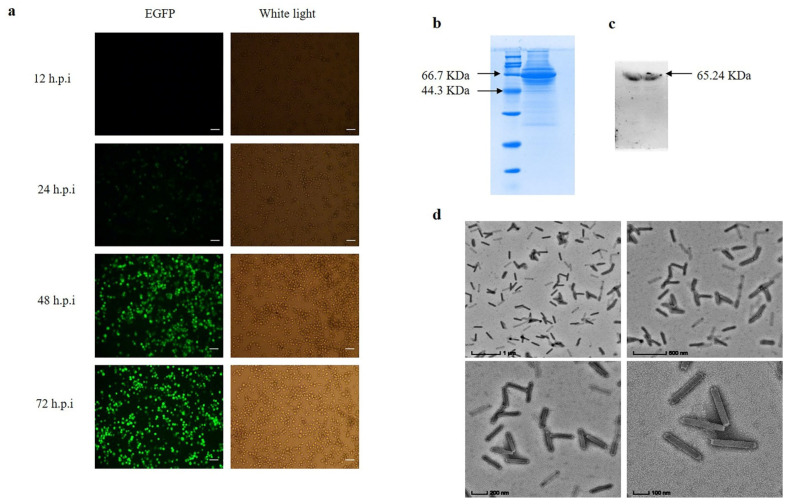
Detection of recombinant baculovirus BmBV-egfp-p64-3×flag-gp64sp. (**a**) Fluorescence gradually increases in BmN cells infected with recombinant baculovirus at 12–72 h.p.i (bar = 100 µm). (**b**) SDS-PAGE of BmBV-egfp-p64-3×flag-gp64sp. (**c**) Western blotting of BmBV-egfp-p64-3×flag-gp64sp (Flag antibody). (**d**) Detection of BmBV-egfp-p64-3×flag-gp64sp structure by transmission electron microscope (bar = 1 µm, 500 nm, 200 nm, 100 nm).

The recombinant baculovirus was passed for three generations to obtain virulence-stabilized progeny baculovirus. The green fluorescence of infected BmN cells gradually stabilized at 48–72 h (Figure 4a). The recombinant baculovirus capsid protein GP64 was detected by SDS-PAGE and Western-Blot, and the results showed successful expression of GP64 protein (Figure 4b,c). The recombinant baculovirus structure was examined by transmission electron microscopy, and the results showed that the baculovirus was structurally intact (Figure 4d).

### 3.2. Co-Immunoprecipitation

BmN cells were infected with the recombinant baculovirus, and the baculovirus infestation was determined by identifying the green fluorescence by inverted fluorescence microscopy (Figure 1b). A 50 mL volume of BmN cells infected with the recombinant baculovirus was then enriched with 2 mL of PBS at 72 h.p.i. Collection of samples at different stages of co-immunoprecipitation was performed for qualitative analysis. Western-blotting results showed that the lysed cells had contained the recombinant baculovirus proteins and the magnetic bead-eluted samples contained protein complexes that interacted with the recombinant baculovirus proteins (Figure 1c).

The recombinant baculovirus was then injected into Dazao silkworm, and the affected silkworms were identified by disease symptoms and green fluorescence targeting the silkworm body (Figure 2b). The epithelium of infected silkworms was collected for liquid nitrogen grinding. As with cell samples, qualitative analysis of each stage of co-immunoprecipitation was performed. The Western blotting results showed that the epithelium of affected silkworm had contained the recombinant baculovirus proteins and the magnetic bead-eluted samples had contained the protein complexes that interacted with the recombinant baculovirus proteins (Figure 2c).

### 3.3. LC-MS/MS Analysis

**Figure 5:**
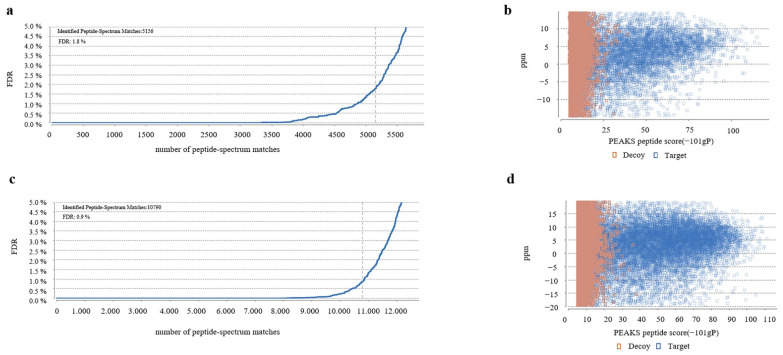
LC -MS/MS analysis of BmN sample and silkworm sample. (**a**) The number of peptide-spectrum matches of the BmN sample. (**b**) PEAKS peptide score of BmN sample. (**c**) The number of peptide-spectrum matches of silkworm sample. (**d**) PEAKS peptide score of silkworm sample.

The magnetic bead eluate was analyzed by LC-MS/MS. A total of 5156 matched peptides were identified in the cells, with a false discovery rate of 1.8% (Figure 5a). Peptide-spectrum matches were mainly concentrated at 0–5 ppm (Figure 5b). Lastly, 845 proteins were matched in the Uniprot protein database for *B. mori* species (Table S1).

A total of 10,790 matched peptides were identified in the silkworm samples, and the false discovery rate was 0.9% (Figure 5c). The peptide-spectrum matches were also mainly concentrated in 0–5 ppm (Figure 5d). A total of 1368 proteins were matched *B. mori* species in the Uniprot protein database (Table S2).

### 3.4. Bioinformatic Analysis of Gene Ontology and KEGG

**Figure 6:**
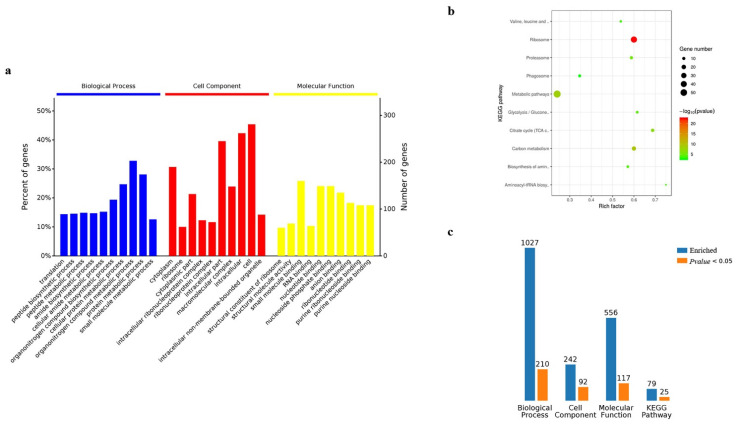
Analysis of Gene Ontology and KEGG for BmN sample. (**a**) Gene Ontology. (**b**) KEGG. (**c**) The summary of Gene Ontology and KRGG regarding enriched counts.

Gene ontology analysis was performed on the 845 candidate proteins obtained from cells. Regarding enrichment in the biological process in cells, the largest proportion was found to be enriched in the metabolic processes of organic nitrogen compounds, followed by the protein metabolic processes. Cellular component analysis found that the largest proportion was enriched in proteins of cell components, followed by proteins of intracellular components, while molecular function analysis indicated that the largest proportion was enriched in proteins involved in the binding function of small molecules, followed by nucleotide-binding proteins (Figure 6a). The KEGG pathway enrichment analysis reported the top ten signaling pathways with significant levels (*p* < 0.05), and the results showed that the 845 candidate proteins were mainly involved in the signaling pathways of cellular metabolism, followed by signaling pathways of ribosome metabolism (Figure 6b). By gathering the results from GO and KEGG analysis in the candidate proteins obtained from cells, the results indicated that 1027 proteins were identified in the biological process, 210 of which were with significantly enriched; cell component enriched 242 proteins, 92 of which were significant; molecular function enriched 556 proteins, 117 of which were significant; Kegg pathway enriched 79 proteins, 25 of which were significant (Figure 6c) (Table S3).

**Figure 7:**
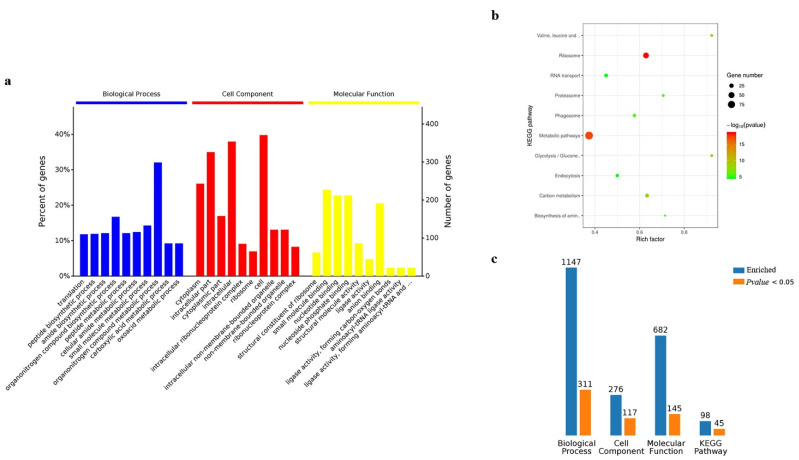
Analysis of Gene Ontology and KEGG for silkworm sample. (**a**) Gene Ontology. (**b**) KEGG. (**c**) The summary of Gene Ontology and KRGG regarding enriched counts.

The results of GO and KEGG analysis in the silkworm samples were similar to those of BmN cells, however, there were differences in the number of proteins (Figure 7a,b). The biological process enriched 1147 proteins, 311 of which were significant; cell component enriched 276 proteins, 117 of which were significant; molecular function enriched 682 proteins, 145 of which were significant; Kegg pathway enriched 98 proteins, 45 of which were significant (Figure 7c) (Table S4).

### 3.5. dsRNA Interferes with the Expression of FABP1, PIC, and SEC61

**Figure 8:**
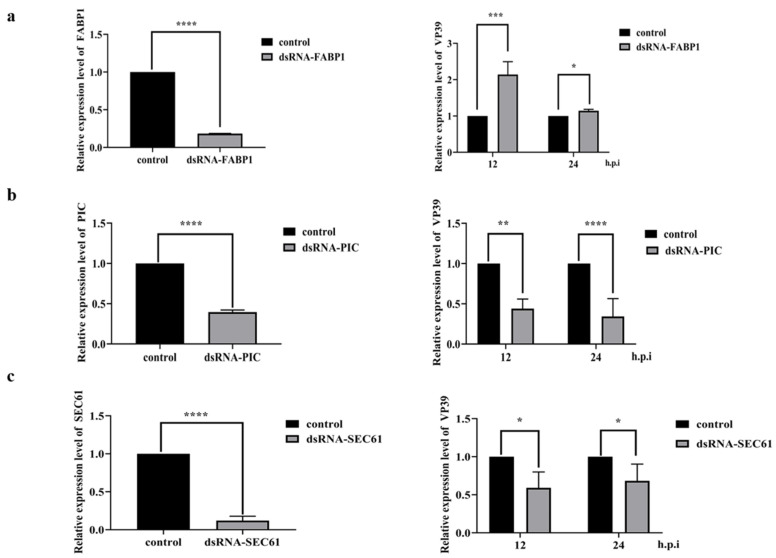
Effects of FABP1, PIC, and SEC61 on the proliferation of BmNPV. (**a**) The RNAi of the dsRNA-FABP decreases the expression level of FABP1 in a dose-dependent manner at 24 h (*p* < 0.0001), The dsRNA-EGFP is transfected as the control. Relative expression levels of the VP39 in the dsRNA-FABP1 transfected BmN cells at 12 h.p.i (*p* < 0.001) and 24 h.p.i (*p* < 0.05) by the rBmNPV. (**b**) The RNAi of the dsRNA-PIC decreases the expression level of PIC in a dose-dependent manner at 24 h (*p* < 0.0001), The dsRNA-EGFP is transfected as the control. Relative expression levels of the VP39 in the dsRNA-PIC transfected BmN cells at 12 h.p.i (*p* < 0.01) and 24 h.p.i (*p* < 0.0001) by the rBmNPV. (**c**) The RNAi of the dsRNA-SEC61 decreases the expression level of SEC61 in a dose-dependent manner at 24 h (*p* < 0.0001), The dsRNA-EGFP is transfected as the control. Relative expression levels of the VP39 in the dsRNA-FABP1 transfected BmN cells at 12 h.p.i (*p* < 0.05) and 24 h.p.i (*p* < 0.05) by the rBmNPV.

Combined with bioinformatics analysis, we selected some significant proteins in the Kegg pathway to investigate their effects on BmNPV replication. These included cellular retinoic acid binding protein FABP1 (ID: Q2QEH2), phosphate carrier protein PIC (ID: Q1HPL2) and transport protein SEC61 (ID: Q19AA9). The dsRNA fragments of FABP1, PCI, and SEC61 were synthesized with the primers shown in Table 1. The results of qPCR showed that down-regulation of FABP1 mRNA level resulted in up-regulation of the relative expression of baculovirus nucleocapsid protein VP39 at 12 h.p.i and 24 h.p.i (Figure 8a), while down-regulation of PIC mRNA level resulted in down-regulation of the relative expression of VP39 at 12 h.p.i and 24 h.p.i (Figure 8b). In addition, down-regulation of SEC61 mRNA level resulted in down-regulation of the relative expression of VP39 at 12 h.p.i and 24 h.p.i (Figure 8c). These results demonstrate that PCI, SEC61 protein together might promote BmNPV replication in the host, while FABP1 protein likely inhibits BmNPV replication in the host.

## 4. Discussion

Viruses are obligate parasites. Upon their invasion of the host and subsequent replication, they are met by interactions with host proteins [30,31]. In this study, we reconstructed the budding viral particle BV of BmNPV using the multi-bac baculovirus expression system and conducted co-immunoprecipitation experiments with the BV as bait to investigate the interaction between viral proteins and host proteins during the invasion and replication of BmNPV. LC-MS/MS analysis revealed a total of 845 and 1368 candidate proteins were obtained in cells and silkworm samples, respectively. Among them, 476 candidate proteins were detected in both cells and silkworm body samples.

There are several approaches for identifying the interaction between viruses and hosts. Feng et al. obtained the protein SINAL10, which interacts with the baculovirus capsid protein GP64, by using a yeast two-hybrid and baculovirus library screening method [25], while Dong et al. obtained the BmREEPa protein by proteomic differential analysis of two cell lines, BmN-SWU1 and BmN-SWU2, which interacts with GP64 protein through the BmPTCHD protein [27]. In the present study, BmREEPa protein was also obtained by screening in cells and silkworm samples. In terms of the time of viral infection, Xue et al., confirmed by transcriptomic analysis that BmNPV reached a maximum interaction of viral proteins with host proteins at 24 h.p.i [32]. In this study, we collected samples for co-immunoprecipitation experiments after the baculovirus infection stabilized at 72 h. Therefore, further optimization of the sample collection time would allow increasing amount of protein.

Viruses can interact directly with host proteins or indirectly through other proteins. The receptor protein for BmNPV has not been identified. The capsid protein GP64 of BmNPV plays an important role in its invasion of the host. The current research shows that the proteins that may interact with the GP64 protein are as follows. (1) Phospholipids, TANI et al., treated human hepatoma cells HepG2 with phospholipase C and then co-incubated with recombinant baculovirus Ac64-CAluc and AcVSVG-CAluc, respectively. The expression of fluorescein was used to determine whether the virus entered the host cells. The results showed a dose-dependent expression level of the luciferase gene in the Ac64-CAluc experimental group, while no significant changes were observed in the AcVSVG-CAluc experimental group [33]. Chernomordik et al., treated host insect cells with phospholipase A, which affected the composition of their membrane phospholipids. In addition, the formation of syncytia or lack thereof was used to determine whether the host cells were infected or not [34]. These results suggest that phospholipids on the cell surface play an important role in both baculovirus infection of insect cells and transduction of mammalian cells. (2) Cholesterol, it is essential for animal life activities, accounting for about 30% of animal cell membranes, and its main function is to maintain membrane stability and regulate membrane fluidity. The cholesterol recognition domain (CRAC) is required to mediate viral endocytosis via lattice proteins. Luz-Madrigal et al., found that the GP64 gene contains a cholesterol recognition domain and that baculovirus infection was significantly reduced when host cell cholesterol was inhibited with MβCD compared to the control group [35]. Hao et al., found that two Cholesterol Recognition Amino Acid Consensus Motifs of GP64 with uncleaved Signal Peptide are required for *B. mori* Nucleopolyhedrovirus infection [36]. (3) Acetyl heparin sulfate, it is a linear polysaccharide present in all animal tissues, which usually occurs as a proteoglycan and binds various protein ligands. Acetyl heparin sulfate has been shown to be a receptor for a variety of viruses, e.g., acetyl heparin sulfate plays an important role in novel coronavirus SARS-CoV-2 infection, especially when the virus is bound to ACE2 [37]. Makkonen et al., showed that a heparin recognition region exists at amino acid sites 271–292 of the baculovirus capsid protein GP64, and in an acidic environment, GP64 binds heparin, especially at pH 6.2, where heparin-binding is highest [38].

**Figure 9:**
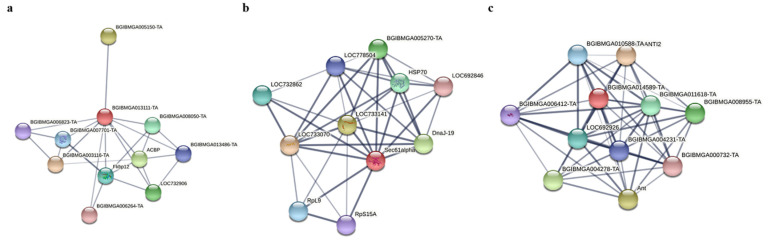
Protein–protein interaction (PPI) analysis of FABP1, PIC and SEC61. (**a**) FABP1. (**b**) SEC61. (**c**) PIC.

Protein–protein interaction (PPI) analysis of FABP1, PIC and SEC61 using STRING (https://string-db.org/, accessed on 15 March 2022). PPI analysis revealed that FABP1 interacts with BGIBMGA013486-TA, which belongs to the ubiquitin-binding enzyme family E2 (Figure 9a), and E3 ubiquitin protein-linked enzymes have been shown to promote the proliferation of BmNPV by facilitating the fusion of its capsid protein GP64 with the host cell membrane. E2 and E3 can interact with each other to transfer ubiquitin to target cells. Therefore, it is speculated that FABP1 may have a competitive relationship with E3, which in turn inhibits the replication of BmNPV. SEC61 plays an important role as a transporter protein with ribosomal proteins and is mainly involved in protein translocation across membranes (Figure 9b). It may be involved in protein translocation in BmNPV replication. PIC belongs to the mitochondrial carrier (TC 2.A.29) family, which is mainly involved in mitochondrial activities, including ATP synthesis coupled proton transport, and may provide energy for the replication of BmNPV (Figure 9c).

## 5. Conclusions

In summary, the present study obtained a large amount of protein data related to BmNPV invasion and replication. This provides a protein data reference for the screening of BmNPV receptors and the study of proteins that play a key role in the replication of BmNPV. Moreover, it provides a basis for subsequent studies on FABP1, SEC61, and PIC proteins.

## Figures and Tables

**Table 1:** Primers used in this study.

Primer Name	Primer Sequence
3×flag-gp64sp-F	5′-aagaattcatgggtaagcgctattgttttatatg-3′
3×flag-gp64sp-R	5′-aagtcgacttaatattgtctagtattacggtt-3′
p64-F	5′-aatcgatctgagcgtccgtgttcatgatcccgttt-3′
p64-R	5′-aaggatcccatcttgcttgtgtgttccttattga-3′
egfp-F	5′-aacccgggatggtgagcaagggcgaggag-3′
egfp-R	5′-aactcgagttacttgtacagctcgtccatgcc-3′
dsRNA-FABP1-F	5′-taatacgactcactatagggtggaattcgtaggcaagaaa-3′
dsRNA-FABP1-R	5′-taatacgactcactatagggtggtgcaagtcacgtcctta-3′
dsRNA-PIC-F	5′-taatacgactcactatagggttgggtattcaatgcagggt-3′
dsRNA-PIC-R	5′-taatacgactcactatagggcctggttgagtttggacaca-3′
dsRNA-SEC61-F	5′-taatacgactcactatagggcatacaactgtttgtggccg-3′
dsRNA-SEC61-R	5′-taatacgactcactatagggactaggaagttgccgctgaa-3′
qPCR-FABP1-F	5′-gacttcctccgagaactttgatg-3′
qPCR-FABP1-R	5′-ggatttcaccttagcaccgtca-3′
qPCR-PIC-F	5′-gagaccgcctacacttaccgt-3′
qPCR-PIC-R	5′-gggcttgggcacaacatac-3′
qPCR-SEC61-F	5′-ggtgctaaaatcattgaagttgg-3′
qPCR-SEC61-R	5′-aacagttgtatgataatgaggaggc-3′
qPCR-RP49-F	5′-caggcggttcaagggtcaatac-3′
qPCR-RP49-R	5′-tgctgggctctttccacga-3′

## Data Availability

Protein data were obtained from the Uniprot database (https://www.uniprot.org/, accessed on 5 June 2021). Data analysis is based on QuickGO (https://www.ebi.ac.uk/QuickGO/, accessed on 8 August 2021) and KEGG database (https://www.kegg.jp/, accessed on 20 September 2021).

## Supplementary Materials

The following supporting information can be downloaded at: https://www.mdpi.com/article/10.3390/insects13070575/s1, Table S1: protein data of BmN; Table S2: protein data of silkworms; Table S3: Bioinformatics Analysis of the BmN; Table S4: Bioinformatics Analysis of the silkworms.

## References

[1] Ackermann H.W., Smirnoff W.A. (1983). A morphological investigation of 23 baculoviruses. J. Invertebr. Pathol..

[2] Gomi S., Majima K., Maeda S. (1999). Sequence analysis of the genome of *Bombyx mori* nucleopolyhedrovirus. J. Gen. Virol..

[3] Harrison R.L., Herniou E.A., Jehle J.A., Theilmann D.A., Burand J.P., Becnel J.J., Krell P.J., van Oers M.M., Mowery J.D., Bauchan G.R. (2018). ICTV virus taxonomy profile: Baculoviridae. J. Gen..

[4] Rohrmann G.F. (2019). Baculovirus Molecular Biology.

[5] Jiang L., Xia Q. (2014). The progress and future of enhancing antiviral capacity by transgenic technology in the silkworm *Bombyx mori*. Insect Biochem. Mol. Biol..

[6] Yang X., Zhang X., Liu Y., Yang D., Liu Z., Chen K., Tang L., Wang M., Hu Z., Zhang S. (2021). Transgenic genome editing-derived antiviral therapy to nucleopolyhedrovirus infection in the industrial strain of the silkworm. Insect Biochem. Mol. Biol..

[7] Li G., Zhou K., Zhao G., Qian H., Xu A. (2019). Transcriptome-wide analysis of the difference of alternative splicing in susceptible and resistant silkworm strains after BmNPV infection. 3 Biotech.

[8] Keddie B.A., Aponte G.W., Volkman L.E. (1989). The pathway of infection of *Autographa californica* nuclear polyhedrosis virus in an insect host. Science.

[9] Miao X.X., Xub S.J., Li M.H., Li M.W., Huang J.H., Dai F.Y., Marino S.W., Mills D.R., Zeng P., Mita K. (2005). Simple sequence repeat-based consensus linkage map of *Bombyx mori*. Proc. Natl. Acad. Sci. USA.

[10] Rahman M.M., Gopinathan K.P. (2004). Systemic and in vitro infection process of *Bombyx mori* nucleopolyhedrovirus. Virus Res..

[11] Clem R.J., Passarelli A.L. (2013). Baculoviruses: Sophisticated pathogens of insects. PLoS Pathog..

[12] Ji X., Sutton G., Evans G., Axford D., Owen R., Stuart D.I. (2010). How baculovirus polyhedra fit square pegs into round holes to robustly package viruses. EMBO J..

[13] Funk C.J., Braunagel S.C., Rohrmann G.F., Miller L.K. (1997). Baculovirus Structure. The Baculoviruses.

[14] Braunagel S.C., Summers M.D. (2007). Molecular biology of the baculovirus occlusion-derived virus envelope. Curr. Drug Targets.

[15] Wang P., Granados R. (1997). An intestinal mucin is the target substrate for a baculovirus enhancin. Proc. Natl. Acad. Sci. USA.

[16] Blissard G.W. (1996). Baculovirus-insect cell interactions. Cytotechnology.

[17] Blissard G.W., Rohrmann G.F. (1990). Baculovirus diversity and molecular biology. Annu. Rev. Entomol..

[18] Lanier L.M., Volkman L.E. (1998). Actin binding and nucleation by *Autographa californica* M nucleopolyhedrovirus. Virology.

[19] Wang Y., Wang Q., Liang C., Song J., Li N., Shi H., Chen X. (2008). *Autographa californica* Multiple Nucleopolyhedrovirus Nucleocapsid Protein BV/ODV-C42 Mediates the Nuclear Entry of P78/83. J. Virol..

[20] Slack J., Arif B.M. (2006). The Baculoviruses Occlusion-Derived Virus: Virion Structure and Function. Adv. Virus Res..

[21] Wu P., Shang Q., Huang H., Zhang S., Zhong J., Hou Q., Guo X. (2019). Quantitative proteomics analysis provides insight into the biological role of Hsp90 in BmNPV infection in *Bombyx mori*. J. Proteom..

[22] Kvansakul M., Caria S., Hinds M.G. (2017). The Bcl-2 Family in Host-Virus Interactions. Viruses.

[23] Vassilaki N., Frakolaki E. (2017). Virus-host interactions under hypoxia. Microbes Infect..

[24] Wang X., Zhang Y., Fei S., Awais M., Zheng H., Feng M., Sun J. (2021). Heat Shock Protein 75 (TRAP1) facilitate the proliferation of the *Bombyx mori* nucleopolyhedrovirus. Int. J. Biol. Macromol..

[25] Feng M., Kong X., Zhang J., Xu W., Wu X. (2018). Identification of a novel host protein SINAL10 interacting with GP64 and its role in *Bombyx mori* nucleopolyhedrovirus infection. Virus Res..

[26] Dong X., Liu T., Wang W., Pan C., Du G., Wu Y., Pan M., Lu C. (2017). BmNHR96 participate BV entry of BmN-SWU1 cells via affecting the cellular cholesterol level. Biochem. Biophys. Res. Commun..

[27] Dong X., Liu T., Wang W., Pan C., Du G., Wu Y., Adur M., Zhang M., Pan M., Lu C. (2017). Transgenic RNAi of BmREEPa in silkworms can enhance the resistance of silkworm to *Bombyx mori* Nucleopolyhedrovirus. Biochem. Biophys. Res. Commun..

[28] Yao L., Wang S., Su S., Yao N., He J., Peng L., Sun J. (2012). Construction of a baculovirus-silkworm multigene expression system and its application on producing virus-like particles. PLoS ONE.

[29] Zheng H., Wang X., Ren F., Zou S., Feng M., Xu L., Yao L., Sun J. (2018). Construction of a highly efficient display system for baculovirus and its application on multigene co-display. Mol. Genet. Genomics.

[30] Maarouf M., Rai K.R., Goraya M.U., Chen J.L. (2018). Immune Ecosystem of Virus-Infected Host Tissues. Int. J. Mol. Sci..

[31] Cruz L., Buchkovich N.J. (2017). Rerouting the traffic from a virus perspective. Front. Biosci..

[32] Xue J., Qiao N., Zhang W., Cheng R., Zhang X., Bao Y., Xu Y., Gu L., Han J., Zhang C. (2012). Dynamic Interactions between *Bombyx mori* Nucleopolyhedrovirus and Its Host Cells Revealed by Transcriptome Analysis. J. Virol..

[33] Tani H., Nishijima M., Ushijima H., Miyamura T., Matsuura Y. (2001). Characterization of cell-surface determinants important for baculovirus infection. Virology.

[34] Chernomordik L., Leikina E., Cho M., Zimmerberg J. (1995). Control of baculovirus gp64-induced syncytium formation by membrane lipid composition. J. Virol..

[35] Luz-Madrigal A., Asanov A., Camacho-Zarco A.R., Sampieri A., Vaca L. (2013). A cholesterol recognition amino acid consensus domain in GP64 fusion protein facilitates anchoring of baculovirus to mammalian cells. J. Virol..

[36] Hao B., Nan W., Xu Y., Liu L., Liu N., Huang J. (2021). Two Cholesterol Recognition Amino Acid Consensus Motifs of GP64 with Uncleaved Signal Peptide Are Required for *Bombyx mori* Nucleopolyhedrovirus Infection. Microbiol. Spectr..

[37] Clausen T.M., Sandoval D.R., Spliid C.B., Pihl J., Perrett H.R., Painter C.D., Narayanan A., Majowicz S.A., Kwong E.M., McVicar R.N. (2020). SARS-CoV-2 infection depends on cellular heparan sulfate and ACE2. Cell.

[38] Makkonen K., Turkki P., Laakkonen J.P., Ylä-Herttuala S., Marjomäki V., Airenne K.J. (2013). 6-*O*- and *N*-Sulfated Syndecan-1 Promotes Baculovirus Binding and Entry into Mammalian Cells. J. Virol..

